# Phase distribution of spliceosomal introns: implications for intron origin

**DOI:** 10.1186/1471-2148-6-69

**Published:** 2006-09-08

**Authors:** Hung D Nguyen, Maki Yoshihama, Naoya Kenmochi

**Affiliations:** 1 Frontier Science Research Center, University of Miyazaki 5200 Kihara, Kiyotake, Miyazaki 889-1692, Japan

## Abstract

**Background:**

The origin of spliceosomal introns is the central subject of the introns-early versus introns-late debate. The distribution of intron phases is non-uniform, with an excess of phase-0 introns. Introns-early explains this by speculating that a fraction of present-day introns were present between minigenes in the progenote and therefore must lie in phase-0. In contrast, introns-late predicts that the nonuniformity of intron phase distribution reflects the nonrandomness of intron insertions.

**Results:**

In this paper, we tested the two theories using analyses of intron phase distribution. We inferred the evolution of intron phase distribution from a dataset of 684 gene orthologs from seven eukaryotes using a maximum likelihood method. We also tested whether the observed intron phase distributions from 10 eukaryotes can be explained by intron insertions on a genome-wide scale. In contrast to the prediction of introns-early, the inferred evolution of intron phase distribution showed that the proportion of phase-0 introns increased over evolution. Consistent with introns-late, the observed intron phase distributions matched those predicted by an intron insertion model quite well.

**Conclusion:**

Our results strongly support the introns-late hypothesis of the origin of spliceosomal introns.

## Background

The origin of spliceosomal introns – "extra" DNA sequences that disrupt the coding regions in nuclear genes of eukaryotes – is still a mystery. Since the evolution of introns is closely related to the evolution of eukaryotic genomes, understanding the origin of introns is vital for understanding the evolution of eukaryotes. There are currently two opposing theories of intron origin. The introns-early theory proposes that introns already existed at the progenote (i.e., the last common ancestor of prokaryotes and eukaryotes) to facilitate the construction of the first genes [1-4]. The introns-late theory, on the other hand, holds that genes at the progenote were intronless, similar to those in present-day prokaryotes, and introns were gained late, after the emergence of eukaryotes [5-7]. There has been no decisive resolution to the debate, and each of these theories has supporting arguments that have not been satisfactorily disproved.

Introns can be located in one of three phases: phase-0, -1, and -2 introns are defined as introns located before the first, after the first, and after the second nucleotide of a codon, respectively. The phase of an intron is conserved during evolution, because a variation in intron phase is possible only through simultaneous mutations that alter the 5' and 3' ends of the intron in a complementary manner [8]. The distribution of intron phases is non-uniform: phase-0 introns occur most frequently and phase-2 introns occur least frequently [8-10].

The introns-early theory explains the non-uniform distribution by speculating that 35% of modern introns are ancient, i.e., existed at the progenote to facilitate the assembly of the first genes [4,11]. Since exons are remnants of primordial minigenes, most of these ancient introns must lie in phase-0, resulting in the current excess of phase-0 introns. However, this theory does not satisfactorily explain why phase-1 introns are more common than phase-2 introns. In contrast, the introns-late theory proposes that the nonuniformity of intron phase distribution may have arisen from nonrandom intron insertion [7]. Introns have been proposed to be inserted only into a fixed sequence pattern, termed a "proto-splice site" [12]. Several potential patterns for proto-splice sites have been proposed, for example MAG|R [12]; G|G, AG|G, AG|GT [10]; and MAG|GT [13,14]. (In these patterns, M is A or C, R is A or G, and the vertical line represents the intron insertion site.) However, there is still no clear evidence that the observed distributions of intron phase are caused by intron insertions [10,15].

In this paper, we tested the introns-early and introns-late theories using two independent approaches: (i) by inferring the evolution of intron phase distribution and (ii) by retesting whether intron phase distribution reflects the nonrandomness of intron insertions. The results show that there is a general trend over evolution toward increasing the preponderance of phase-0 introns and that the observed phase distribution of introns can be indeed explained by an intron insertion model. Consequently, our results seem to support the explanation provided by the introns-late theory for the nonuniformity of intron phase distribution.

## Results

### Inference of the evolution of intron phase distribution

**Figure 1:**
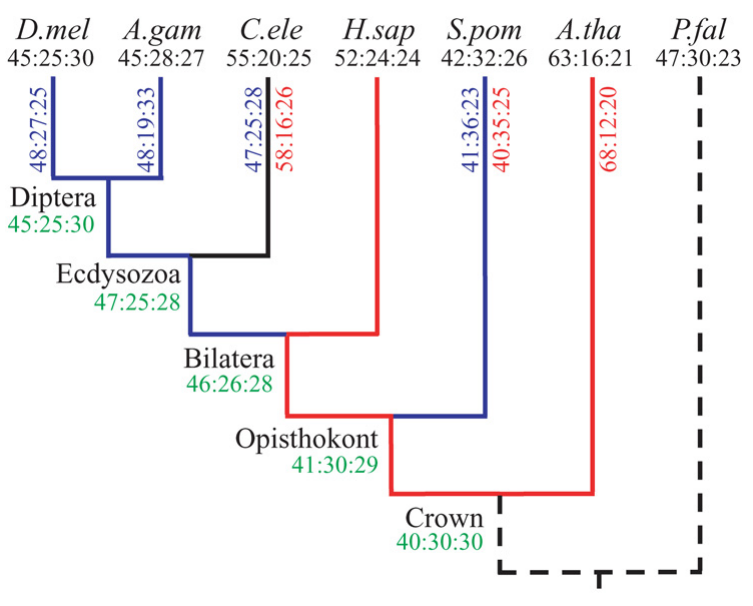
The evolution of intron phase distribution in the 684-ortholog dataset. Phase distributions (phase-0:phase-1:phase-2) of introns in modern species (known) are in black. Phase distributions of introns in ancestors (estimated) are in green. Phase distributions of gained and lost introns (estimated) are in red and blue, respectively. All phase distributions are based on events of >90% probability of occurrence. Where there is no such event, phase distributions are not shown. Branches that experienced >1.5 gains per loss are shown in red and those that experienced >1.5 losses per gain are in blue. D. mel, *D. melanogaster*; A. gam, *A. gambiae*; C. ele, *C. elegans*; H. sap, *H. sapiens*; S. pom, *S. pombe*; A. tha, *A. thaliana*; P. fal, *P. falciparum*.

Figure 1 shows the evolution of intron phase distribution inferred from intron patterns in conserved regions of 684 gene orthologs from seven eukaryotes using an assumed ecdysozoa tree and the maximum likelihood method of estimating rates of intron gains and losses. There is a general trend toward an increasing proportion of phase-0 introns caused by gained introns. For two branches, one from the crown ancestor to *Arabidopsis thaliana *and the other from the ecdysozoa ancestor to *Caenorhabditis elegans*, the differences between phase distributions of gained introns and ancestral introns are statistically significant (*P *= 8.3 × 10^-16 ^and 1.8 × 10^-5^, respectively). In contrast, differences between the phase distributions of lost introns and ancestral introns are not statistically significant for any branch that has data for lost introns. Our result for the evolution of intron phase distribution thus suggests that the nonuniformity of intron phase distribution is more likely to be due to the nonrandomness of intron insertions.

### Compilation of a genome-wide dataset

In order to test the introns-late prediction that intron phase distribution is non-uniform, we compiled a dataset from the entire genomes of 10 eukaryotes (Table 1). These 10 species were chosen because they cover a broad range of evolutionary distance and their genomes are well annotated. In this dataset, the average number of introns per gene varies from 1.0 in *Schizosaccharomyces pombe *to 8.1 in *Homo sapiens*. The GC content of the coding regions in the genomes ranges from 24% in *Plasmodium falciparum *to 56% in *Neurospora crassa*, and the distribution of phase-0 introns ranges from 38.2% in *N. crassa *to 57.6% in *A. thaliana*. In all species the intron phase distributions show an obvious pattern of phase-0 > phase-1 > phase-2; the only exception is *A. thaliana*, in which the distribution of phase-2 introns is slightly larger than that of phase-1 introns. These results are consistent with previously published results (e.g., ref. [10]).

**Table 1:** Statistical information of the genome-wide dataset.

Organism	# of genes	# of introns	Avg. introns	%GC	Phase distribution (%)		
					
					P-0	P-1	P-2
*N. crassa*	8817	15856	1.80	56.0	38.2	34.1	27.7
*D. melanogaster*	8932	27135	3.04	53.7	42.5	31.8	25.7
*H. sapiens*	11058	89508	8.09	52.3	45.4	31.2	23.4
*F. graminearum*	8168	18695	2.29	51.8	38.6	34.4	27.0
*C. neoformans*	5603	26945	4.81	51.0	41.6	30.7	27.7
*X. tropicalis*	7793	61999	7.96	47.2	46.7	30.3	23.0
*A. thaliana*	9734	52856	5.43	44.1	57.6	20.6	21.8
*C. elegans*	11128	60110	5.40	43.1	47.8	26.5	25.7
*S. pombe*	3791	3924	1.04	39.4	42.2	31.2	26.6
*P. falciparum*	3828	6127	1.60	23.6	44.8	32.6	22.6

The 10 eukaryotes are arranged in descending order of GC contents (%) from top to bottom. P-0, phase-0; P-1, phase-1; P-2, phase-2.

### Prediction of intron phase distribution for the all-pattern model

**Figure 2:**
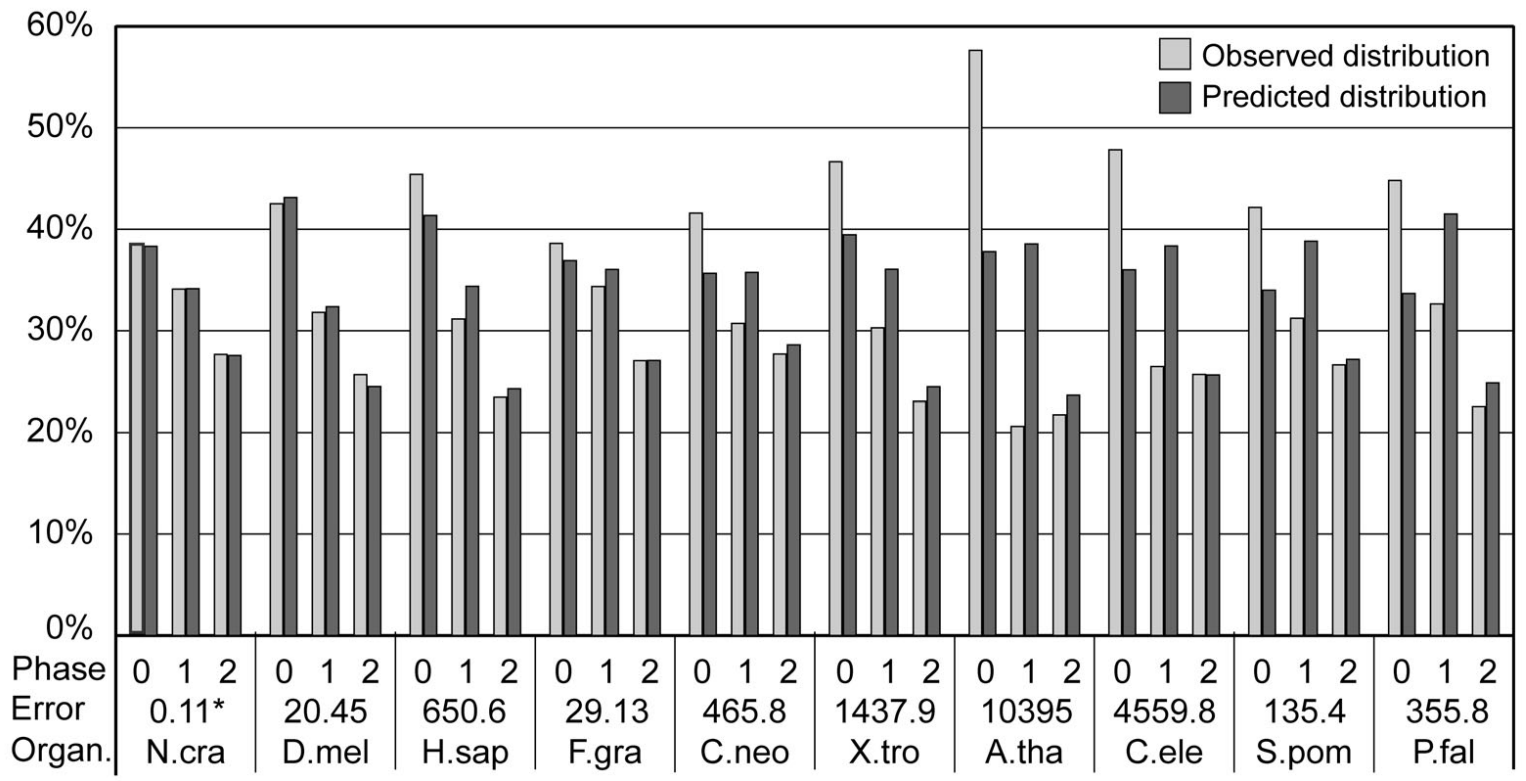
Intron phase distributions predicted using the all-pattern intron insertion model. Error is measured as the χ^2 ^value between the observed and predicted intron phase distributions. The 10 eukaryotes are arranged in descending order of GC contents (%) from left to right. N. cra, *N. crassa*; D. mel, *D. melanogaster*; H. sap, *H. sapiens*; F. gra, *F. graminearum*; C. neo, *C. neoformans*; X. tro, *X. tropicalis*; A. tha, *A. thaliana*; C. ele, *C. elegans*; S. pom, *S. pombe*; P. fal, *P. falciparum*. * Not significant at the *P *< 0.05 level. All other comparisons were significant.

Figure 2 shows the intron phase distributions predicted by an intron insertion model (hereafter, the all-pattern model) in which introns can be inserted into any sequence pattern, but are inserted into different patterns with different frequencies. The predicted intron phase distributions matched the observed ones quite well for GC-rich species with GC content >50% (e.g., *N. crassa *and *Drosophila melanogaster*), but did not match for GC-poor species with GC content <50% (e.g., *A. thaliana *and *C. elegans*). For all GC-poor species, the largest errors in prediction occurred in phase-0 and phase-1 introns; the proportions of phase-0 introns were underestimated whereas those of phase-1 introns were overestimated. Note that although most *Xenopus tropicalis *introns are shared with *H. sapiens *introns (unpublished data), the GC content is 5% lower and the prediction error is much larger in *X. tropicalis*. Based on this observation, we speculated that the larger prediction errors in GC-poor species may be due to higher mutation rates.

### Inference of the GC content and intron density in the RP gene dataset

**Figure 3:**
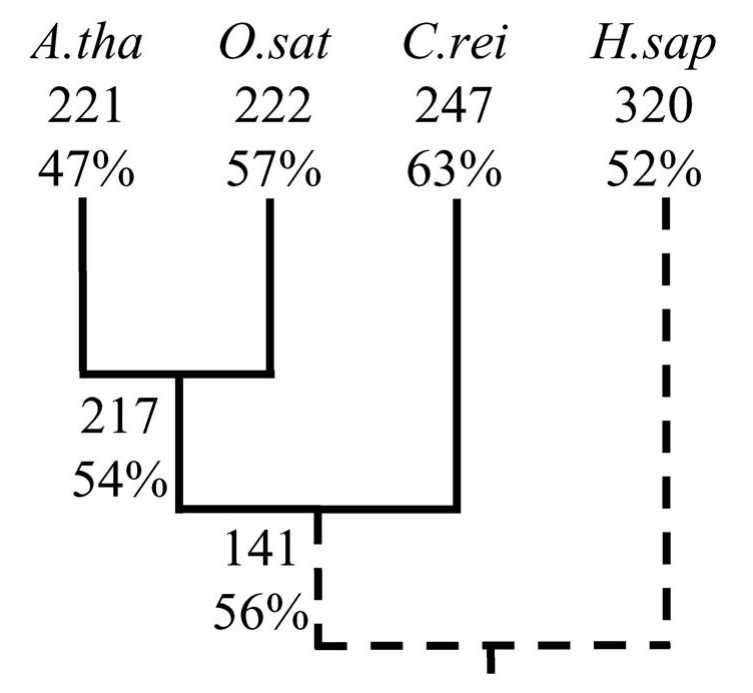
The evolution of intron density and GC content in 79 orthologs of ribosomal protein genes. The numbers show intron densities whereas the percentages show GC contents. A. tha, *A. thaliana*; O. sat, *O. sativa*; C. rei, *C. reinhardtii*; H. sap, *H. sapiens*.

To test our speculation that the prediction errors were due to high mutation rates, we compiled a smaller dataset containing 79 orthologs of ribosomal protein (RP) genes from four species: *A. thaliana*, *Oryza sativa*, *Chlamydomonas reinhardtii*, and *H. sapiens*, and inferred the evolution of GC content and intron density (Figure 3). The three plant species were chosen because *A. thaliana *had the largest prediction error using the all-pattern model (Figure 2). The outgroup *H. sapiens *was chosen due to its nearly neutral (52%) GC content and its high density of introns. The analysis indicated that 98% of *A. thaliana *introns already existed in its last common ancestor with *O. sativa*, and the inferred GC content for this ancestor was 54%. The result suggests that the large reduction in GC content (from 54% to 47%) in *A. thaliana *is likely to be the main cause for its large prediction error. (Note that although the GC content of RP genes is somewhat different from the average GC content in each whole genome, this does not affect the result significantly, as only the relative differences are important here.) It is possible that when introns are inserted, the exon junctions surrounding introns are subjected to a much lower mutation rate than the average mutation rate in the genes of fast-evolving species due to the need for efficient splicing. Consequently, the intron phase distributions predicted using current sequences in fast-evolving species would not match the observed data.

### Prediction of intron phase distribution with mutation correction

**Figure 4:**
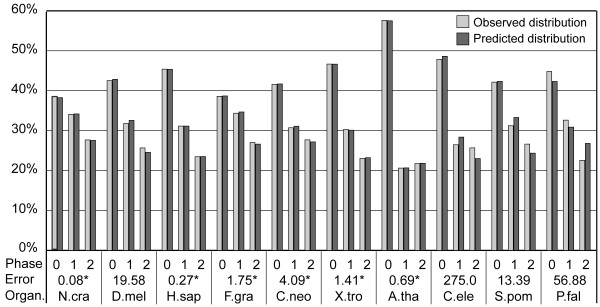
Intron phase distributions predicted using the all-pattern intron insertion model and mutation correction. Error is measured as the average of χ^2 ^values between the observed and predicted intron phase distributions in 20 simulations for mutation correction. The 10 eukaryotes are arranged in descending order of GC contents (%) from left to right. N. cra, *N. crassa*; D. mel, *D. melanogaster*; H. sap, *H. sapiens*; F. gra, *F. graminearum*; C. neo, *C. neoformans*; X. tro, *X. tropicalis*; A. tha, *A. thaliana*; C. ele, *C. elegans*; S. pom, *S. pombe*; P. fal, *P. falciparum*. * Not significant at the *P *< 0.05 level. All other comparisons were significant.

To accommodate this source of error in fast-evolving species, we proposed a simple model for mutation correction and used it to re-predict the intron phase distributions for all species in the genome-wide dataset (Figure 4). The best mutation rates (the rate at which the prediction error is smallest), the corresponding GC contents, the predicted intron phase distributions, the prediction errors, and the standard deviations for all species are provided in Table 2. As shown in Figure 4, the differences between the predicted intron phase distributions and the observed ones were now not statistically significant (i.e., *P *> 0.05) for *H. sapiens*, *N. crassa*, *Fusarium graminearum*, *Cryptococcus neoformans*, *A. thaliana*, and *X. tropicalis*. There are several lines of evidence for the validity of our mutation correction model. First, for *A. thaliana*, the GC content at the best mutation rate was 57.6% (Table 2 [see Additional file 1]), a value very close to the inferred 54% of the last common ancestor of *A. thaliana *and *O. sativa *in the 79 orthologs of RP genes (Figure 3). It is possible that this value was the average GC content of *A. thaliana *during the period when most of its introns were gained. Second, the best prediction errors and GC contents of *H. sapiens *and *X. tropicalis *were close to each other, in agreement with the fact that most *H. sapiens *introns are shared with those of *X. tropicalis *and their divergence is quite recent (unpublished data). (The small difference between the two inferred best GC contents is likely due to difference in the GC content of the second bases of codons, because our model does not correct for mutations at these bases.) Third, the inferred best GC contents of the two other animals: *D. melanogaster *and *C. elegans *were also very close to those of *H. sapiens *and *X. tropicalis*. Finally, our result suggests that the human genome is evolving toward decreasing its GC content, consistent with the result of Meunier and Duret [16].

**Table 2:** Prediction of intron phase distributions using the all-pattern intron insertion model and mutation correction.

Organism	Best *mrate*	Best %GC	Phase distribution (%)			Error	σ
					
			P-0	P-1	P-2		
*N. crassa*	-0.004	55.9	38.2	34.2	27.6	0.08*	0.001
*D. melanogaster*	-0.011	53.5	42.8	32.6	24.6	19.58	0.055
*H. sapiens*	+0.178	55.0	45.4	31.1	23.5	0.27*	0.066
*F. graminearum*	+0.163	54.3	38.7	34.7	26.6	1.75*	0.024
*C. neoformans*	+0.503	59.9	41.7	31.1	27.2	4.09*	0.125
*X. tropicalis*	+0.279	52.3	46.7	30.1	23.2	1.41*	0.138
*A. thaliana*	+0.632	57.6	57.5	20.7	21.8	0.69*	0.147
*C. elegans*	+0.539	54.6	48.6	28.4	23.0	274.98	2.260
*S. pombe*	+0.406	49.2	42.3	33.3	24.4	13.39	0.099
*P. falciparum*	+0.390	34.7	42.3	30.9	26.8	56.88	0.347

"Best *mrate*" shows the best mutation rate. "Best %GC", "phase distribution (%)", and "error" are averages of 20 simulations for mutation correction at the best mutation rate. Error is measured as the χ^2 ^value between the observed and predicted intron phase distributions. "σ" shows the standard deviation of error in 20 simulations. P-0, phase-0; P-1, phase-1; P-2, phase-2.

* Not significant at the *P *< 0.05 level. All other comparisons were significant.

## Discussion

The introns-early theory explains the excess of phase-0 introns by predicting that a fraction of present-day introns are ancient and these introns were in phase-0. If this explanation is correct, the excess of phase-0 introns should generally decrease during eukaryotic evolution as new introns are inserted into random positions. A direct test of the explanation of the introns-early theory for the excess of phase-0 introns is therefore to infer the evolution of intron phase distribution from observed data. This test was first performed by Roy *et al*. [17]. Using a dataset of 280 ancient genes (unpublished), they divided the present-day introns into two categories: lineage-specific introns and widely phylogenetically distributed introns, which are thought to be rough estimates of recently gained introns and ancestral introns, respectively. They found that the presumed ancestral introns had a stronger phase-0 bias than the lineage-specific introns (Table 3 of ref. [17]). In contrast, our results (Figure 1) show a general trend over evolution toward an increase in the excess of phase-0 introns. We believe that this discrepancy is more likely due to different datasets than to different classification methods because when a classification method similar to that used in ref. [17] was applied to the current dataset, a stronger phase-0 bias in lineage-specific introns was obtained [18]. Another reason for this discrepancy may be that all of the 280 gene families in the dataset used in ref. [17] are ancient, and these gene families may show a different pattern of evolution of intron phase distribution than younger gene families. However, when we used a smaller dataset of 79 RP gene families – all of which are believed to be ancient – from the same seven species studied here [19], the result was still inconsistent with that in ref. [17] (data not shown).

Sverdlov *et al*. [18] suggested that the stronger phase-0 bias in lineage-specific introns than in widely distributed introns refuted the explanation of the introns-early hypothesis. However, it should be stressed that this conclusion cannot always be drawn from this result: The explanation of introns-early may still be correct even when lineage-specific introns have stronger phase-0 bias than widely distributed introns. Consider the following example: suppose a species has 200 current introns with a phase distribution of 100:50:50, and 100 of these are widely distributed introns with a phase distribution of 40:30:30. Therefore, the species also has 100 lineage-specific introns with a phase distribution of 60:20:20. We suppose further that all 100 lineage-specific introns were gained recently and there are also 100 introns specific to this species that have been lost. If the phase distribution of the lost introns is 40:30:30, the phase distribution of ancestral introns will be 80:60:60, which has less phase-0 bias than the current introns. However, if the phase distribution of lost introns is 80:10:10, the phase distribution of ancestral introns will be 120:40:40, which has more phase-0 bias than the current introns. Thus, no decisive conclusion can be reached by comparing intron phase distributions between lineage-specific introns and widely distributed introns. In contrast, by using the maximum likelihood method to infer a set of most reliable events (>90% probability of occurrence), we were able to estimate the intron phase distribution at each ancestral node.

Our result for the evolution of intron phase distribution suggests that the excess of phase-0 introns is more likely to be caused by the nonrandomness of intron gains. However, all previous studies failed to prove this at a satisfactory level [10,15]. Therefore, we decided to re-test this prediction on a large scale using genome-wide data from 10 model species. We first used the fixed-pattern intron insertion model, in which introns are inserted only into proto-splice sites, and our experimental results (data not shown) were consistent with previous results [10], in which the intron phase distributions predicted from the distributions of four potential proto-splice sites (G|G, AG|G, AG|GT, and MAG|R) did not match the observed ones.

Another model of intron insertion has been proposed in which introns are either randomly inserted into sequences but with different rates of fixation or are preferentially inserted into a consensus sequence [14,20,21]. We therefore tested the all-pattern intron insertion model, in which introns can be inserted into any pattern of sequences but are inserted into different patterns with different frequencies. Since the frequencies of intron insertion may vary from species to species, these frequencies were obtained from the observed data separately for each species. The results (Figure 2) show that the model predicted intron phase distributions well in GC-rich species but not in GC-poor species. Analysis of a smaller dataset of 79 orthologs of RP genes shows that higher mutation rates are very likely the main cause for the higher prediction errors in GC-poor species (Figure 3). Therefore, we proposed a simple model for mutation correction and used it to predict intron phase distributions for all species again. As expected, the predicted intron phase distributions now matched the observed data for both GC-rich and GC-poor species, with differences in six out of ten species that were not statistically significant (Figure 4 and Table 2).

Although the predicted intron phase distributions of four remaining species (*D. melanogaster*, *C. elegans*, *S. pombe*, and *P. falciparum*) account quite well for the observed distributions (Figure 4), their differences were still statistically significant. It is possible that the assumption of not changing amino acid sequences in our mutation correction model did not fully compensate for the mutation effect in *S. pombe *and *P. falciparum*, as they have very low GC contents. The larger errors in *D. melanogaster *and *C. elegans *may be partly due to the nonuniformity of intron losses, because both species suffered from high rates of intron loss after their divergence from *H. sapiens *[22]. Moreover, since other factors such as annotation mistakes on exon/intron structures may also affect the results, we should not put too much weight on statistical tests. Therefore, we conclude that the all-pattern intron insertion model may explain intron phase distributions even when statistical equivalence is not reached.

The intron phase distributions are lineage-specific and may be affected by two factors: changes in DNA sequences and changes in intron insertion frequencies. The latter may reflect changes in the efficiency with which the splicing machinery splices out introns. When the intron insertion frequencies learned from *H. sapiens *were used to predict *N. crassa *sequences, the predicted intron phase distribution was 44:32:24, much closer to the distribution observed in *H. sapiens *(45:31:24) than in *N. crassa *(38:34:28). This indicates that the change in intron insertion frequencies has stronger effect on the intron phase distribution than the change in DNA sequences.

## Conclusion

The debate surrounding introns-early versus introns-late remains vigorous [23,24]. We previously provided two lines of support for the introns-late view: there is no general trend over evolution toward decreasing intron density [[22], but also see ref. [25]]; and there was no clear case of intron position conservation in a set of 25 cytoplasmic RP genes of archaeal origin and mitochondrial RP genes of bacterial origin, which are thought to have diverged at the progenote [26]. In this paper, we have provided two more lines of support for introns-late from analyses of intron phase distribution: the current excess of phase-0 introns is due to the excess of phase-0 among gained introns and not to the remnants of minigenes; and the all-pattern intron insertion model can explain the observed intron phase distributions in various species. These results should help to resolve the long-standing but important debate about the origin of spliceosomal introns.

## Methods

### Inference of the evolution of intron phase distribution

Koonin's group [27] compiled intron patterns in the conserved regions of 684 gene orthologs from eight eukaryotes, *D. melanogaste*r, *A. gambiae*, *H. sapiens, C. elegans*, *Saccharomyces cerevisiae*, *S. pombe*, *A. thaliana*, and *P. falciparum*. We used this database for our analysis, but excluded *S. cerevisiae *due to its sparse intron distribution. Following our previous analysis [22], we assumed the ecdysozoa tree for these species and applied our maximum likelihood method to infer rates of intron gains and losses as well as the distribution of introns in the last common ancestor of these species. These parameters were then used to infer the most reliable events (>90% confidence) for intron gain and loss along each branch and for intron presence at each ancestor. Phase distributions were then calculated for these events using the phase information for each intron pattern. Note that our method [22] assumes the same model of intron evolution with the method of Csűrös [28] but the implementation details are different [29,30].

### Compilation of a genome-wide dataset

We downloaded data about the genomes of six eukaryotes (*H. sapiens*, *D. melanogaster*, *C. elegans*, *S. pombe*, *A. thaliana*, and *P. falciparum*) from NCBI [31], three eukaryotes (*N. crassa*, *F. graminearum*, and *C. neoformans*) from BROAD Institute's Fungal Genome Initiative website [32], and *X. tropicalis *from the JGI Eukaryotic Genomics website [33]. For all genomes except *X. tropicalis*, gene structures were built using annotation. For *X. tropicalis*, there was no annotation for gene structures, so we first used the cDNA sequences as input to the BLAST program [34] to query against the DNA sequences. Then the DNA region covering the query result of each cDNA sequence was extracted and the SIM4 program [35] was used to reconstruct the exon/intron structure. If SIM4 failed to reconstruct the exon/intron structure of a gene (i.e., either match ratio or cover ratio <100%), this gene was discarded. An *ad hoc *program was written in the C programming language to automate the construction of gene structures.

The genes of each genome were then subjected to a purging process to remove redundancy by using a criterion of < 20% amino acid identity. If a pair of genes had identity ≥20%, the one with fewer introns was removed. Another *ad hoc *program, which makes use of the program ALIGN [36] for calculating the identity of a pair of amino acid sequences, was written in C to automate the purging process.

### Prediction of intron phase distribution for the all-pattern model

In the all-pattern intron insertion model, we used patterns of 5-bp length, with 3 bp upstream and 2 bp downstream of the splice sites. The number of patterns, *N*, is therefore 1,024 (= 4^5^). Let *O*_*i *_(*i *= 1..*N*) be the count of pattern *i *among all observed splice sites; *C*_*i *_(*i *= 1..*N*) be the count of pattern *i *in all coding regions; and *D*_*ij *_(*i *= 1..*N*, *j *= 0..2) be the count of pattern *i *appearing at phase *j *in all coding regions. The preference of intron insertion in pattern *i *is proportional to *E*_*i *_= *O*_*i*_/*C*_*i *_and the frequency of intron insertion in pattern *i*, *F*_*i*_, is calculated by:

Fi=Ei/∑i=1NEi     (1)
 MathType@MTEF@5@5@+=feaafiart1ev1aaatCvAUfKttLearuWrP9MDH5MBPbIqV92AaeXatLxBI9gBaebbnrfifHhDYfgasaacH8akY=wiFfYdH8Gipec8Eeeu0xXdbba9frFj0=OqFfea0dXdd9vqai=hGuQ8kuc9pgc9s8qqaq=dirpe0xb9q8qiLsFr0=vr0=vr0dc8meaabaqaciaacaGaaeqabaqabeGadaaakeaacqWGgbGrdaWgaaWcbaGaemyAaKgabeaakiabg2da9maalyaabaGaemyrau0aaSbaaSqaaiabdMgaPbqabaaakeaadaaeWbqaaiabdweafnaaBaaaleaacqWGPbqAaeqaaaqaaiabdMgaPjabg2da9iabigdaXaqaaiabd6eaobqdcqGHris5aaaakiaaxMaacaWLjaWaaeWaceaacqaIXaqmaiaawIcacaGLPaaaaaa@4022@

Then the expected number of phase-*j *introns, *P*_*j*_, is calculated by:

Pj=∑i=1NDij×Fi     (2)
 MathType@MTEF@5@5@+=feaafiart1ev1aaatCvAUfKttLearuWrP9MDH5MBPbIqV92AaeXatLxBI9gBaebbnrfifHhDYfgasaacH8akY=wiFfYdH8Gipec8Eeeu0xXdbba9frFj0=OqFfea0dXdd9vqai=hGuQ8kuc9pgc9s8qqaq=dirpe0xb9q8qiLsFr0=vr0=vr0dc8meaabaqaciaacaGaaeqabaqabeGadaaakeaacqWGqbaudaWgaaWcbaGaemOAaOgabeaakiabg2da9maaqahabaGaemiraq0aaSbaaSqaaiabdMgaPjabdQgaQbqabaGccqGHxdaTcqWGgbGrdaWgaaWcbaGaemyAaKgabeaaaeaacqWGPbqAcqGH9aqpcqaIXaqmaeaacqWGobGta0GaeyyeIuoakiaaxMaacaWLjaWaaeWaceaacqaIYaGmaiaawIcacaGLPaaaaaa@4398@

Finally, the expected percentage of phase-*j *introns, *W*_*j*_, is calculated by:

Wj=PjP0+P1+P2×100(%)     (3)
 MathType@MTEF@5@5@+=feaafiart1ev1aaatCvAUfKttLearuWrP9MDH5MBPbIqV92AaeXatLxBI9gBaebbnrfifHhDYfgasaacH8akY=wiFfYdH8Gipec8Eeeu0xXdbba9frFj0=OqFfea0dXdd9vqai=hGuQ8kuc9pgc9s8qqaq=dirpe0xb9q8qiLsFr0=vr0=vr0dc8meaabaqaciaacaGaaeqabaqabeGadaaakeaacqWGxbWvdaWgaaWcbaGaemOAaOgabeaakiabg2da9maalaaabaGaemiuaa1aaSbaaSqaaiabdQgaQbqabaaakeaacqWGqbaudaWgaaWcbaGaeGimaadabeaakiabgUcaRiabdcfaqnaaBaaaleaacqaIXaqmaeqaaOGaey4kaSIaemiuaa1aaSbaaSqaaiabikdaYaqabaaaaOGaey41aqRaeGymaeJaeGimaaJaeGimaaJaeiikaGIaeiyjauIaeiykaKIaaCzcaiaaxMaadaqadiqaaiabiodaZaGaayjkaiaawMcaaaaa@4723@

### Inference of the GC content and intron density in the RP gene dataset

We compiled 79 orthologs of RP genes from four eukaryotes: *H. sapiens*, *A. thaliana*, *O. sativa*, and *C. reinhardtii*. The RP genes of *H. sapiens *and *A. thaliana *were taken from the manually curated Ribosomal Protein Gene database [19]. The RP genes of *O. sativa *and *C. reinhardtii *were first collected from the TIGR Rice Genome Annotation website [37] and the JGI Eukaryotic Genomics website [33], respectively, by performing BLAST searches using RP genes of *A. thaliana *as queries. Their gene structures were then manually constructed by using both annotation and the gene structures of *H. sapiens *and *A. thaliana *as references. When a gene of a species existed in multiple copies, the copy with the most introns was used.

Multiple sequence alignments for each of these gene orthologs were built using CLUSTAL W [38], and an *ad hoc *program was written in C to extract an intron presence/absence matrix and the conserved DNA regions of these alignments. The conserved regions were then concatenated together and the DNAML program of the PHYLIP package [39] was used to infer the phylogenetic tree and GC contents of the internal nodes of these four species. The GC contents were based only on inferred sites of >95% confidence. Finally, the intron presence/absence matrix and the phylogenetic tree (with *H. sapiens *as the outgroup) were used as input to our maximum likelihood method [22] to infer the intron evolution for these species.

### Prediction of intron phase distribution with mutation correction

We applied a simple model for mutation correction in which only mutations [see Additional file 2] that change the GC content of a codon but do not affect the translated amino acid are allowed. All positions that allow these mutations are assumed to have the same mutation rate, with positive/negative values meaning that these mutations will happen in the direction that increases/decreases the GC content of a codon. For each mutation rate, the original sequences were randomly mutated using this rate and then the intron phase distribution was predicted using the same protocol for the all-pattern model, but with *C*_*i *_and *D*_*ij *_taken from the mutated sequences instead of the original sequences. The simulated mutation correction was repeated 20 times and the average of the 20 χ^2 ^values between the predicted and observed intron phase distributions was used as the prediction error for the mutation rate. We then searched for the best mutation rate (i.e., the rate at which the prediction error is smallest) in the range (-1, +1) using the Brent search algorithm [40]. Intron phase distribution predicted using the best mutation rate was taken as the output. A program was written in C to automatically perform the prediction of intron phase distribution for the all-pattern intron insertion model, both with and without mutation correction.

## Abbreviations

RP: ribosomal protein

## Authors' contributions

HDN, MY, and NK conceived and designed the research. HDN performed the experiments. HDN, MY, and NK analyzed the data. HDN contributed analysis tools. HDN and NK wrote the paper. All authors read and approved the final manuscripts.

## Supplementary Material

Additional File 1 **Variations of GC content and prediction error as a function of mutation rate in *A. thaliana***. The file shows the variations of GC content and prediction error as a function of mutation rate in *A. thaliana*. Click here for file

Additional File 2 **List of all allowable mutations**. The file lists all allowable mutations used for mutation correction. Click here for file

## References

[B1] Doolittle WF (1978). Genes in pieces: were they ever together?. Nature.

[B2] Blake CCF (1978). Do genes in pieces imply proteins in pieces?. Nature.

[B3] Gilbert W (1987). The exon theory of genes. Cold Spring Harb Symp Quant Biol.

[B4] Roy SW (2003). Recent evidence for the exon theory of genes. Genetica.

[B5] Cavalier-Smith T (1991). Intron phylogeny: a new hypothesis. Trends Genet.

[B6] Palmer JD, Logsdon JM (1991). The recent origins of introns. Curr Opin Genet Dev.

[B7] Logsdon JM (1998). The recent origins of spliceosomal introns revisited. Curr Opin Genet Dev.

[B8] Fedorov A, Suboch G, Bujakov M, Fedorova L (1992). Analysis of nonuniformity in intron phase distribution. Nucleic Acids Res.

[B9] Long M, Rosenberg C, Gilbert W (1995). Intron phase correlations and the evolution of the intron/exon structure of genes. Proc Natl Acad Sci USA.

[B10] Long M, de Souza SJ, Rosenberg C, Gilbert W (1998). Relationship between proto-splice sites and intron phases: evidence from dicodon analysis. Proc Natl Acad Sci USA.

[B11] de Souza SJ, Long M, Klein RJ, Roy SW, Lin S, Gilbert W (1998). Toward a resolution of the introns early/late debate: only phase zero introns are correlated with the structure of ancient proteins. Proc Natl Acad Sci USA.

[B12] Dibb NJ, Newman AJ (1989). Evidence that introns arose at proto-splice sites. EMBO J.

[B13] Qiu WG, Schisler N, Stoltzfus A (2004). The evolutionary gain of spliceosomal introns: sequence and phase preferences. Mol Biol Evol.

[B14] Sverdlov AV, Rogozin IB, Babenko VN, Koonin EV (2004). Reconstruction of ancestral protosplice sites. Curr Biol.

[B15] Ruvinsky A, Eskesen ST, Eskesen FN, Hurst LD (2005). Can codon usage bias explain intron phase distribution and exon symmetry?. J Mol Evol.

[B16] Meunier J, Duret L (2004). Recombination drives the evolution of GC-content in the human genome. Mol Biol Evol.

[B17] Roy SW, Lewis BP, Fedorov A, Gilbert W (2001). Footprints of primordial introns on the eukaryotic genome. Trends Genet.

[B18] Sverdlov AV, Rogozin IB, Babenko VN, Koonin EV (2003). Evidence of splice signal migration from exon to intron during intron evolution. Curr Biol.

[B19] Nakao A, Yoshihama M, Kenmochi N (2004). RPG: the Ribosomal Protein Gene database. Nucleic Acids Res.

[B20] Sadusky T, Newman AJ, Dibb NJ (2004). Exon junction sequences as cryptic splice sites: implications for intron origin. Curr Biol.

[B21] Stoltzfus A (2004). Molecular evolution: introns fall into place. Curr Biol.

[B22] Nguyen HD, Yoshihama M, Kenmochi N (2005). New maximum likelihood estimators for eukaryotic intron evolution. PloS Comput Biol.

[B23] Roy SW, Gilbert W (2006). The evolution of spliceosomal introns: patterns, puzzles and progress. Nat Rev Genet.

[B24] Martin W, Koonin EV (2006). Introns and the origin of nucleus-cytosol compartmentalization. Nature.

[B25] Roy SW, Gilbert W (2005). Rates of intron loss and gain: implications for early eukaryotic evolution. Proc Natl Acad Sci USA.

[B26] Yoshihama M, Nakao A, Nguyen HD, Kenmochi N (2006). Analysis of ribosomal protein gene structures: implications for intron evolution. PloS Genet.

[B27] Rogozin IB, Wolf YI, Sorokin AV, Mirkin BG, Koonin EV (2003). Remarkable interkingdom conservation of intron positions and massive, lineage-specific intron loss and gain in eukaryotic evolution. Curr Biol.

[B28] Csűrös M, McLysaght A, Huson D Likely Scenarios of intron evolution. Proceedings of the Comparative Genomics: RECOMB 2005 International Workshop: 18–20 September 2005; Dublin.

[B29] Csűrös M (2006). On the estimation of intron evolution. PLoS Comput Biol.

[B30] Nguyen HD, Yoshihama M, Kenmochi N (2006). Authors' reply. PLoS Comput Biol.

[B31] NCBI. http://www.ncbi.nlm.nih.gov/.

[B32] Fungal Genome Initiative. http://www.broad.mit.edu/annotation/fgi/.

[B33] JGI Eukaryotic Genomics. http://genome.jgi-psf.org/euk_cur1.html.

[B34] Altschul SF, Madden TL, Schaffer AA, Zhang J, Zhang Z, Miller W, Lipman DJ (1997). Gapped BLAST and PSI-BLAST: a new generation of protein database search programs. Nucleic Acids Res.

[B35] Florea L, Hartzell G, Zhang Z, Rubin GM, Miller W (1998). A computer program for aligning a cDNA sequence with a genomic DNA sequence. Genome Res.

[B36] Myers EW, Miller W (1988). Optimal alignments in linear space. CABIOS.

[B37] TIGR Rice Genome Annotation. http://www.tigr.org/tdb/e2k1/osa1/.

[B38] Thompson JD, Higgins DG, Gibson TJ (1994). CLUSTAL W: improving the sensitivity of progressive multiple sequence alignment through sequence weighting, position-specific gap penalties and weight matrix choice. Nucleic Acids Res.

[B39] Felsenstein J, Churchill GA (1996). A Hidden Markov Model approach to variation among sites in rate of evolution. Mol Biol Evol.

[B40] Press WH, Teukolsky SA, Vetterling WT, Flannery BP (1992). Minimization or maximization of functions. Numerical recipes in C: The art of scientific computing.

