# Supplementary material for: Phase distribution of spliceosomal introns: implications for intron origin
Source: BMC Evol Biol. 2006 Sep 8;6:69. doi: 10.1186/1471-2148-6-69 (PMC1574350; doi:10.1186/1471-2148-6-69)
Supplement: Additional File 1 — Variations of GC content and prediction error as a function of mutation rate in A. thaliana. The file shows the variations of GC content and prediction error as a function of mutation rate in A. thaliana. [file 1471-2148-6-69-S1.pdf]

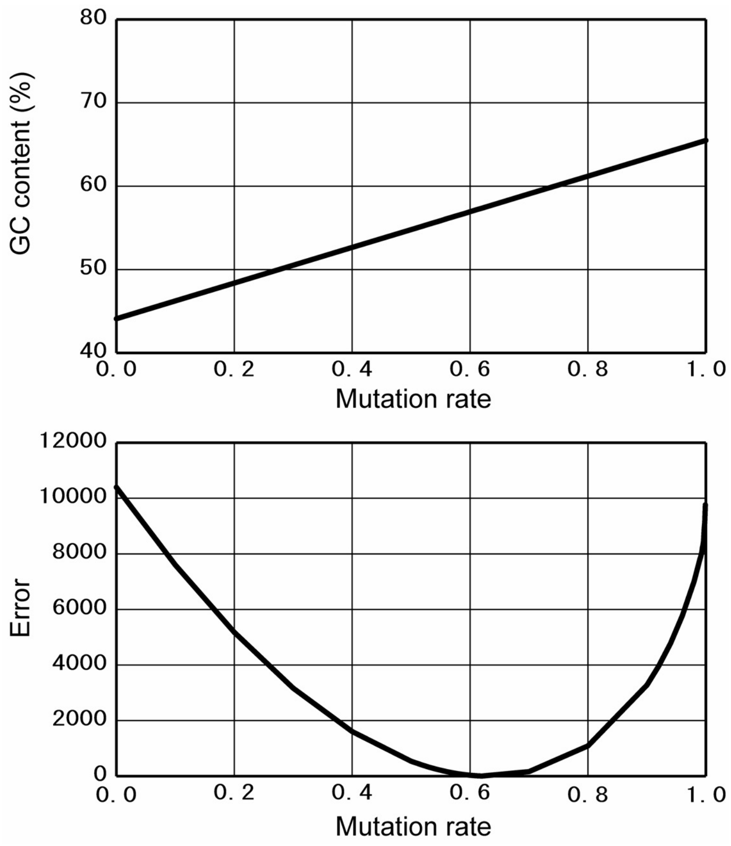

Figure S1. Variations of GC content (upper) and prediction error (lower) as a function of mutation rate in *A. thaliana*.

GC content and error are averages of 20 simulations for mutation correction. Error is measured as the χ^2 value between the observed and predicted intron phase distributions.
