# Supplementary material for: Phase distribution of spliceosomal introns: implications for intron origin
Source: BMC Evol Biol. 2006 Sep 8;6:69. doi: 10.1186/1471-2148-6-69 (PMC1574350; doi:10.1186/1471-2148-6-69)
Supplement: Additional File 2 — List of all allowable mutations. The file lists all allowable mutations used for mutation correction. [file 1471-2148-6-69-S2.pdf]

Table S1. List of all allowable mutations

AA	Mutation	AA	Mutation
L	TTA ↔ CTA	K	AAA ↔ AAG
L	TTG ↔ CTG	S	AGT ↔ AGC
R	AGA ↔ CGA	R	AGA ↔ AGG
R	AGG ↔ CGG	D	GAT ↔ GAC
F	TTT ↔ TTC	E	GAA ↔ GAG
L	TTA ↔ TTG	S	TC{A/T} ↔ TC{G/C}
Y	TAT ↔ TAC	L	CT{A/T} ↔ CT{G/C}
*	TAA ↔ TAG	P	CC{A/T} ↔ CC{G/C}
C	TGT ↔ TGC	R	CG{A/T} ↔ CG{G/C}
H	CAT ↔ CAC	T	AC{A/T} ↔ AC{G/C}
Q	CAA ↔ CAG	V	GT{A/T} ↔ GT{G/C}
I	ATT ↔ ATC	A	GC{A/T} ↔ GC{G/C}
N	AAT ↔ AAC	G	GG{A/T} ↔ GG{G/C}

The original and mutated bases are underlined.
